# Supplementary material for: Structural basis for shape-selective recognition and aminoacylation of a D-armless human mitochondrial tRNA
Source: Nat Commun. 2022 Aug 30;13:5100. doi: 10.1038/s41467-022-32544-1 (PMC9427863; doi:10.1038/s41467-022-32544-1)
Supplement: Supplementary file 1 — Supplementary Information [file 41467_2022_32544_MOESM1_ESM.pdf]

SUPPLEMENTARY INFORMATION

Structural basis for shape-selective recognition and aminoacylation of a D-armless human mitochondrial tRNA

Bernhard Kuhle*, Marscha Hirschi, Lili K. Doerfel, Gabriel C. Lander, Paul Schimmel

* Corresponding author: BK (bkuhle@scripps.edu)

This file includes:

Supplementary Figures 1–12

Supplementary Tables 1-5

Supplementary References

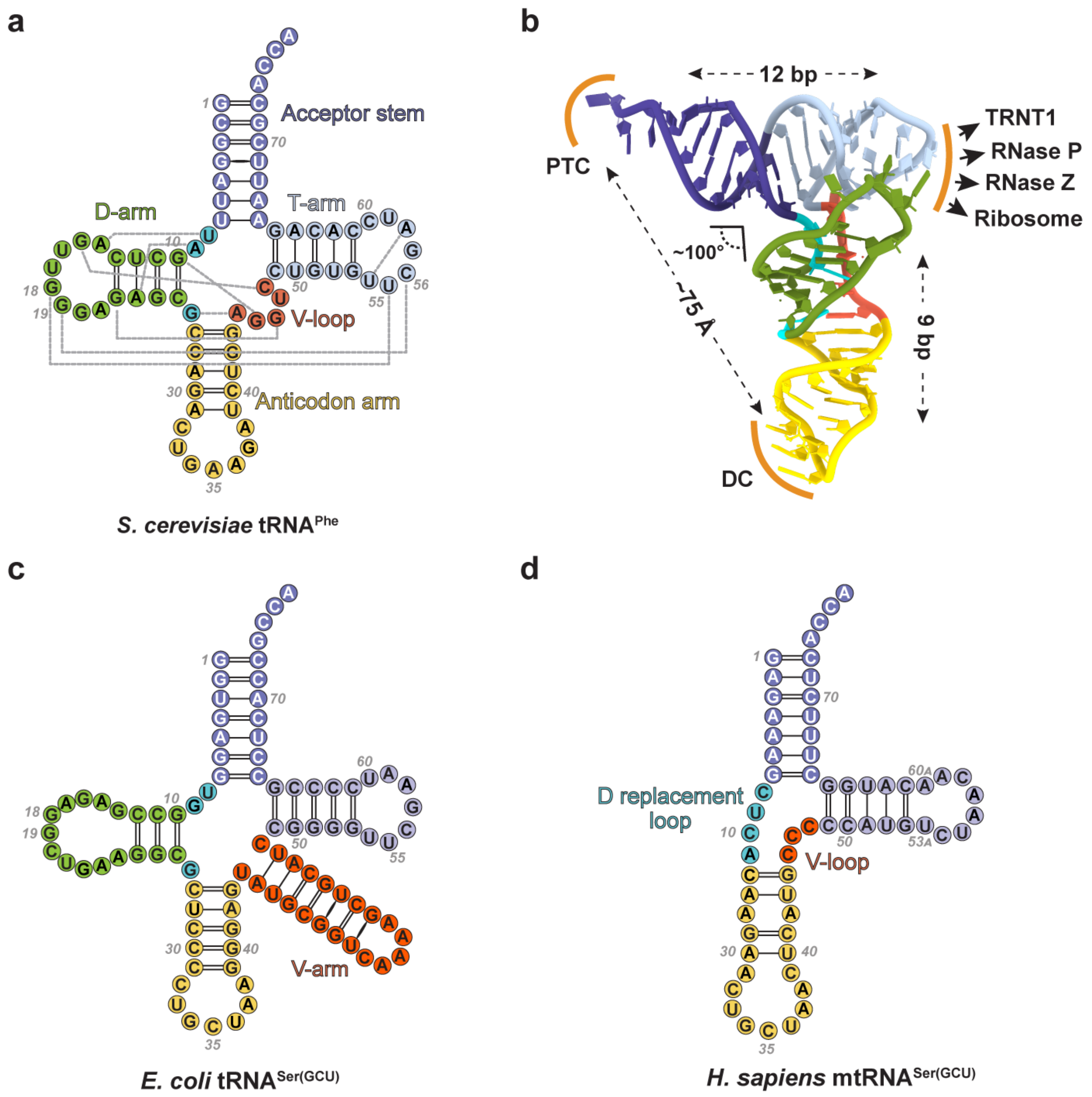

Supplementary Figure 1. Loss of the canonical tRNA structure and serine identity elements in human mtRNA$^{Ser(GCU)}$. Related to Figures 1 and 2. **a.** The cloverleaf structure of *S. cerevisiae* tRNA^{Phe}, representing the canonical secondary structure of prokaryote and eukaryote cytoplasmic tRNAs. Conserved tertiary interactions are indicated as dashed lines. Positions of nucleotides are numbered according to conventional rules[1]. **b.** The L-shaped tertiary fold of *S. cerevisiae* tRNA^{Phe} (PDB 4TRA)[2], showing functional constraints on the canonical tRNA fold as a platform for common interactions with other components of canonical translation machineries, including ribosomal peptidyl-transferase center (PTC) and decoding center (DC). **c.** The *E. coli* tRNA$^{Ser(GCU)}$ containing the long variable arm as major serine identity element embedded into the canonical tRNA fold. **d.** Secondary structure of the highly degenerated human mitochondrial tRNA$^{Ser(GCU)}$.

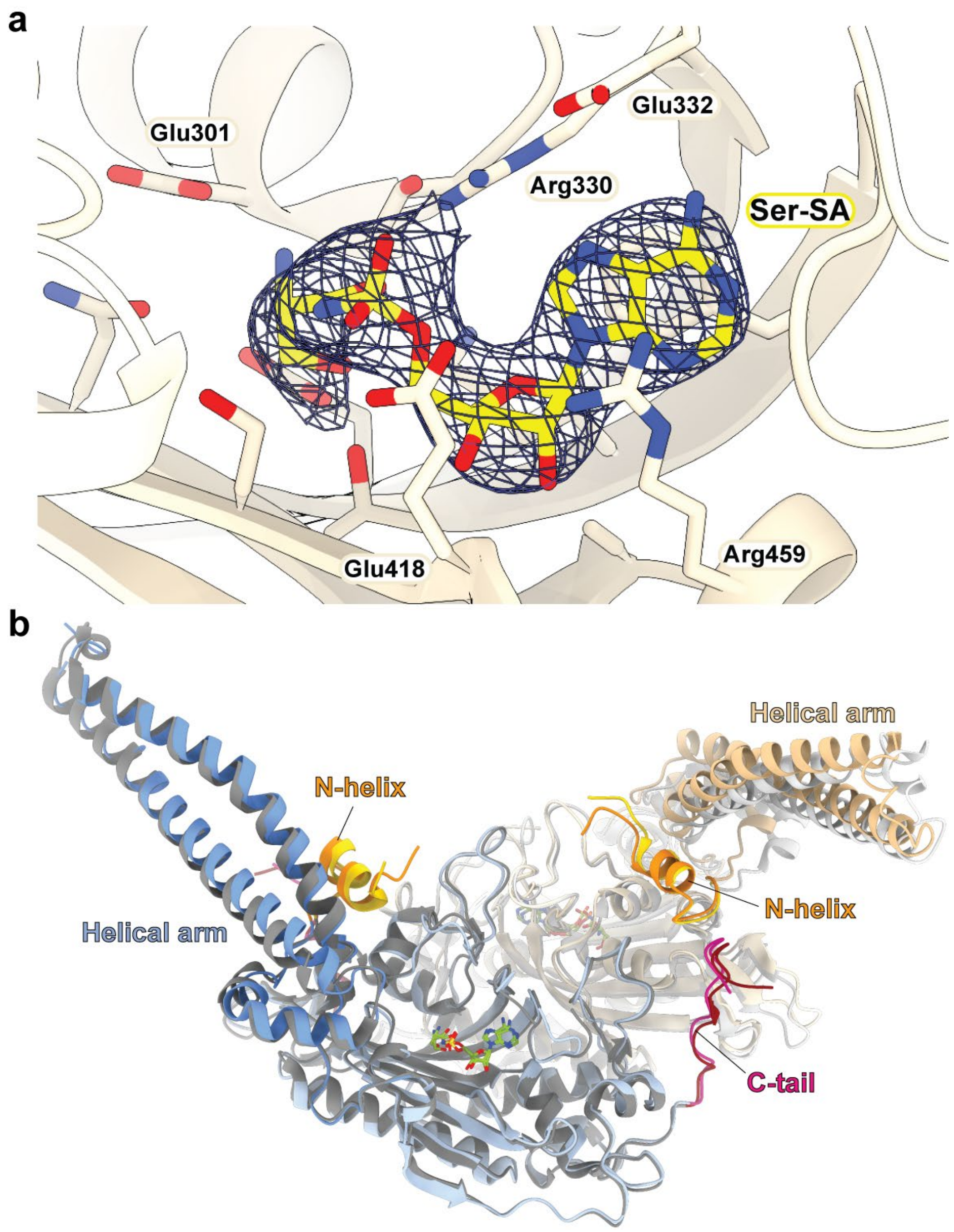

Supplementary Figure 2. Crystal structure of human mSerRS. Related to Fig. 1. **a.** Binding site of SerSA in the crystal structure of human mSerRS with the 2mFo-DFc omit map for the SerSA ligand contoured at 2.5 σ. Residues forming the binding pocket for the seryl-adenylate analog are shown as sticks. **b.** Superposition of human and bovine mSerRSs (PDB 1WLE)[3]. Both mSerRSs are shown in cartoon presentation. Subunits 1 and 2 are respectively colored in blue and wheat for human mSerRS and dark gray and light gray for bovine mSerRS. The mSerRS-specific N-helix and C-tail are respectively highlighted in orange and pink for human mSerRS and in yellow and dark red for bovine mSerRS. SerSA is shown as green sticks in the active sites of both mSerRSs. The sequence identity between the two mSerRS homologues is 87% and their structures superimpose with a root mean square deviation (RMSD) of 0.442 Å over 674 Cα atoms. The largest structural deviations are observed in the two helical arms.

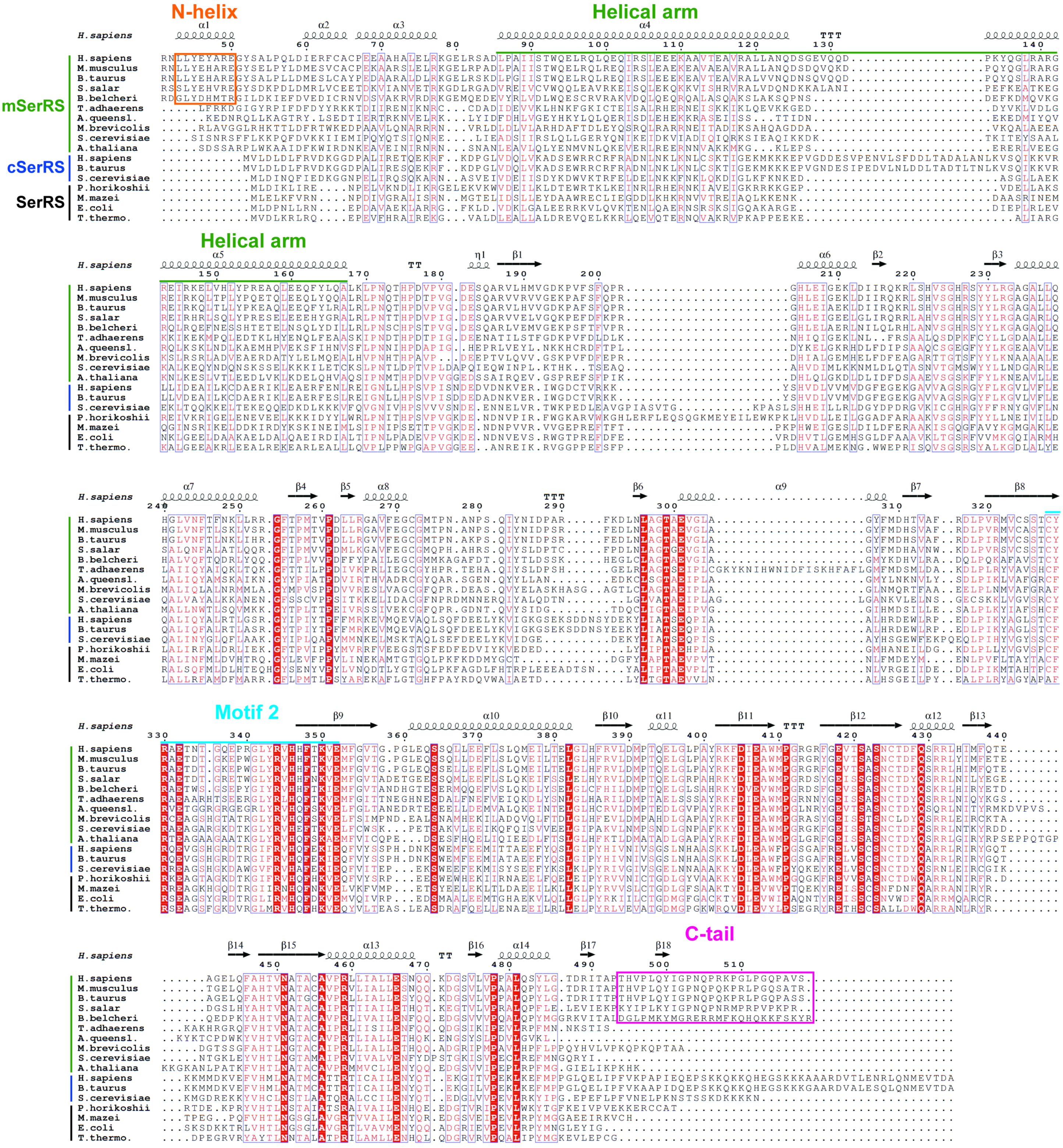

Supplementary Figure 3. Sequence alignment of SerRS variants. Related to Fig. 1. Multiple sequence alignment of mitochondrial (m), eukaryote cytoplasmic (c), and prokaryote SerRSs. Vertebrate mSerRS-specific N- and C-terminal extensions are denoted as N-helix and C-tail, respectively. The alignment was generated with Clustal Omega (https://www.ebi.ac.uk/Tools/msa/clustalo/).

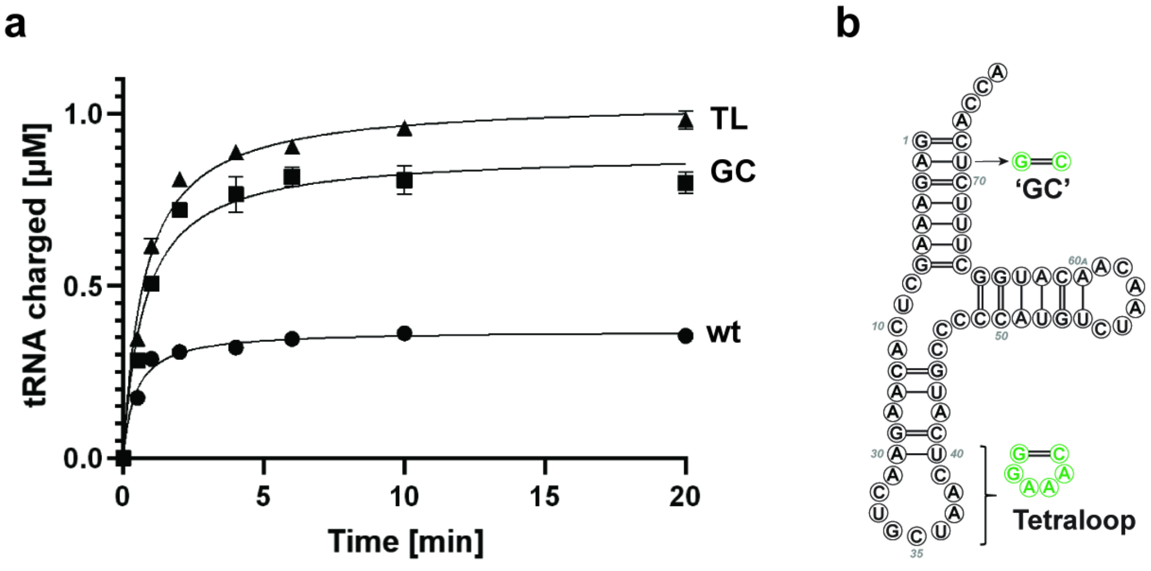

Supplementary Figure 4. Human mtRNA$^{Ser(GCU)}$ variants used for structure and kinetic analysis. Related to Fig. 1. Charging activities (**a**) and secondary structures (**b**) for variants of human mtRNA$^{Ser(GCU)}$ used in this study. Aminoacylation reactions contained 0.25 µM mSerRS and 2 µM tRNA and were carried out at room temperature. Data points represent the mean, and error bars represent the SEM from three independent experiments.

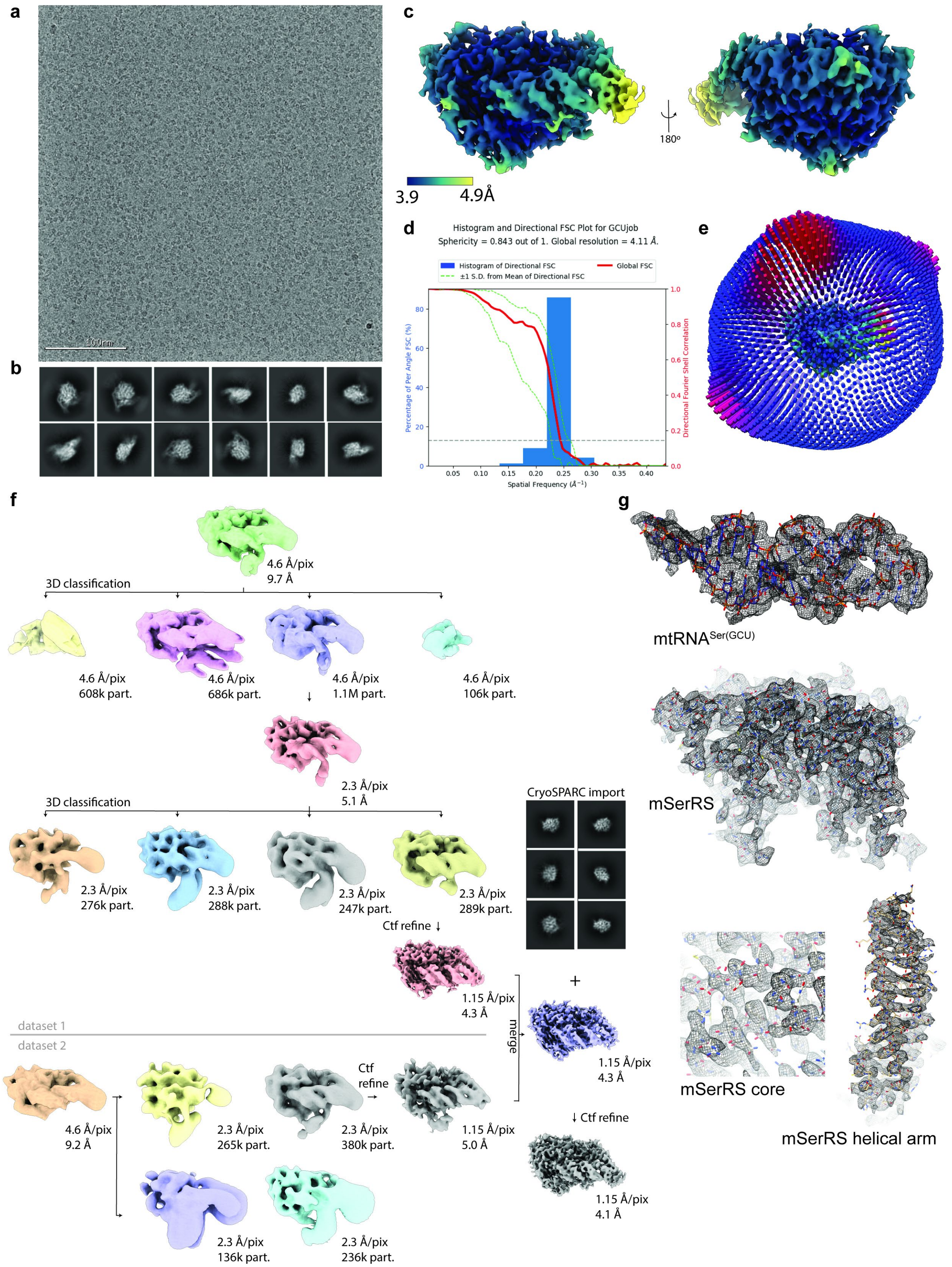

Supplementary Figure 5. Cryo-EM data collection and processing of human mSerRS-mtRNA$^{Ser(GCU)}$. Related to Fig. 1. **a.** Representative micrograph of the specimen. **b.** Selected 2D class averages showing secondary structural information. **c.** Local resolution of the mSerRS-mtRNA$^{Ser(GCU)}$ reconstruction. **d.** Fourier Shell Correlation between the half maps. **e.** Euler distribution of the final reconstruction. **f.** Data processing scheme for mSerRS-mtRNA$^{Ser(GCU)}$. **g.** Components of the mSerRS-mtRNA$^{Ser(GCU)}$ complex fit into the EM density.

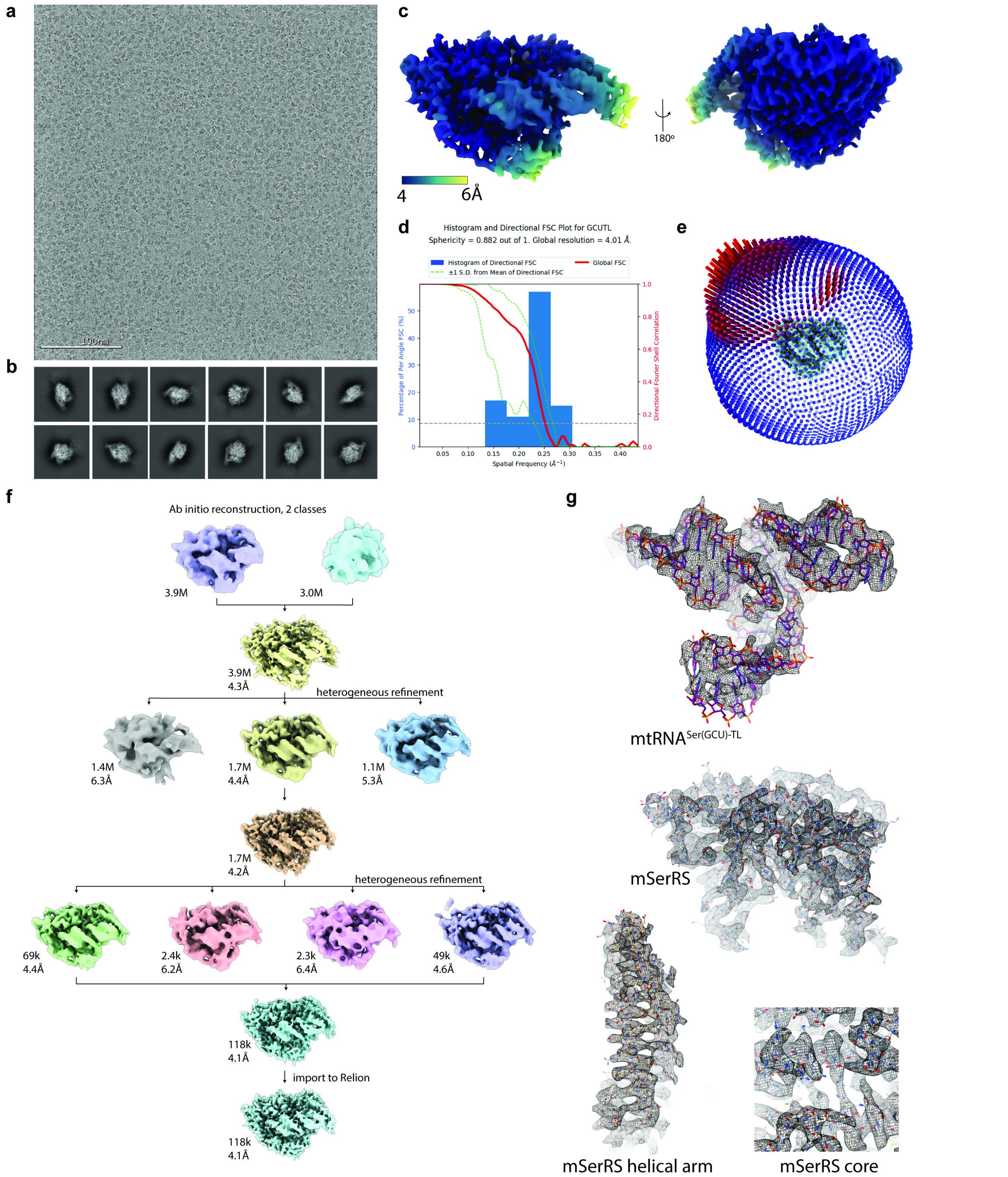

Supplementary Figure 6. Cryo-EM data collection and processing of human mSerRS-mtRNA$^{Ser(GCU)-TL}$. Related to Fig. 1. **a.** Representative micrograph of the specimen tilted to 40 degrees. **b.** Selected 2D class averages showing secondary structure information. **c.** Local resolution of the mSerRS-mtRNA$^{Ser(GCU)-TL}$ reconstruction. **d.** Fourier Shell Correlation between the half maps. **e.** Euler distribution of the final reconstruction. **f.** Data processing scheme for mSerRS-mtRNA$^{Ser(GCU)-TL}$. **g.** Components of the mSerRS-mtRNA$^{Ser(GCU)-TL}$ complex fit into the EM density.

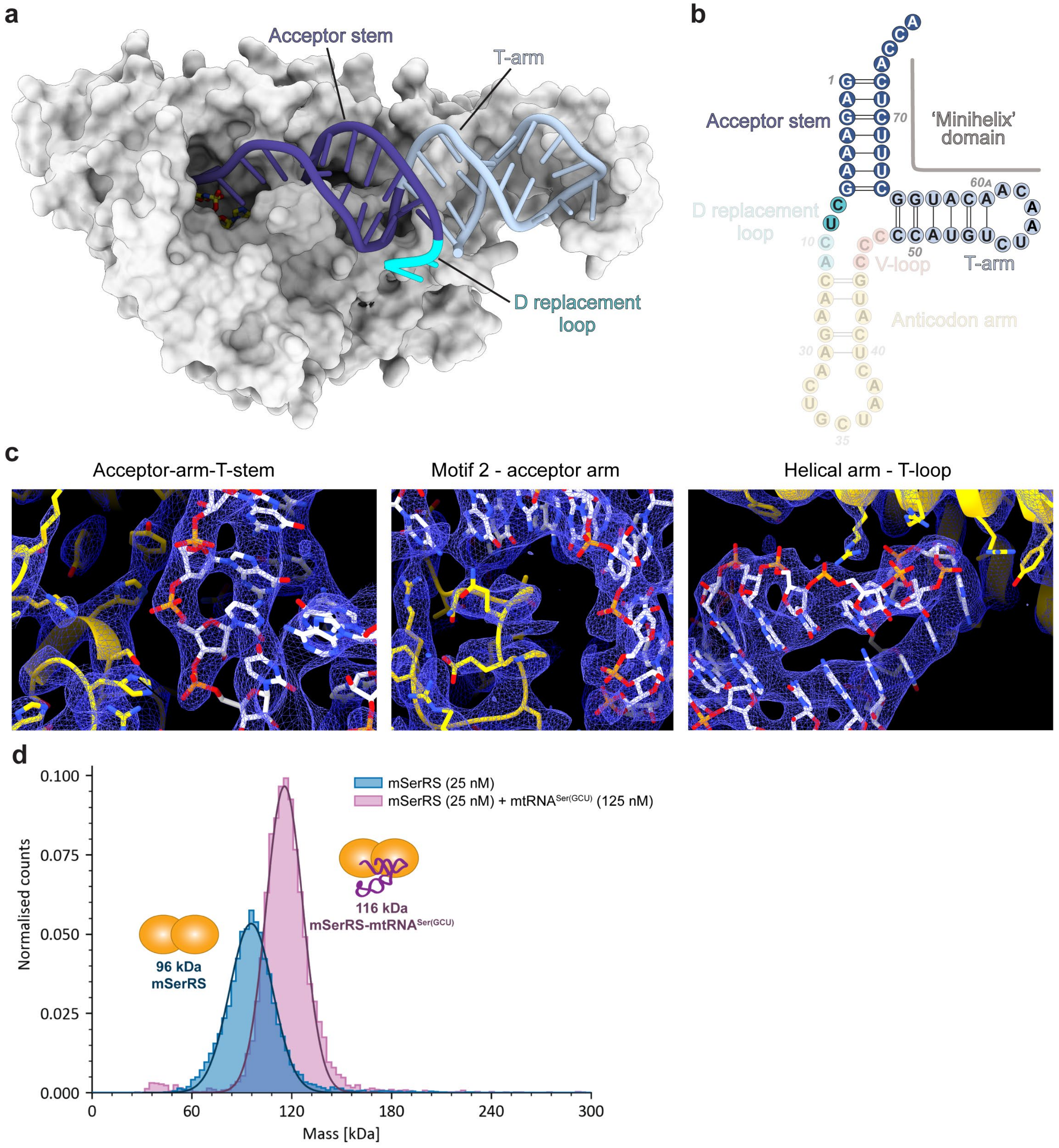

Supplementary Figure 7. Structure of mSerRS bound with full-length mtRNA$^{Ser(GCU)}$ and EM densities. Related to Figures 1-3. **a.** The structure of the mSerRS-tRNA$^{Ser(GCU)}$ complex formed with full-length tRNA. The model contains only the minihelix domain of mtRNA$^{Ser(GCU)}$, consisting of the coaxially stacked acceptor- and T-arms (dark and light blue, respectively). The mSerRS dimer is shown as gray surface. mtRNA$^{Ser(GCU)}$ is shown in cartoon presentation and colored as shown in **b**. **b.** Secondary structure of mtRNA$^{Ser(GCU)}$ highlighting the regions defined in the model of the full-length mtRNA$^{Ser(GCU)}$ shown in **a**. **c**. Close-up views of the EM densities for the mSerRS-mtRNA$^{Ser(GCU)-TL}$ complex shown with the underlying refined atomic models. The protein is colored in yellow, tRNA in light gray. **d.** Mass photometry results for mSerRS-mtRNA$^{Ser(GCU)}$ complex formation. The mSerRS dimer has a calculated molecular mass of 108 kDa, the calculated molecular mass of mtRNA$^{Ser(GCU)}$ is 20.2 kDa.

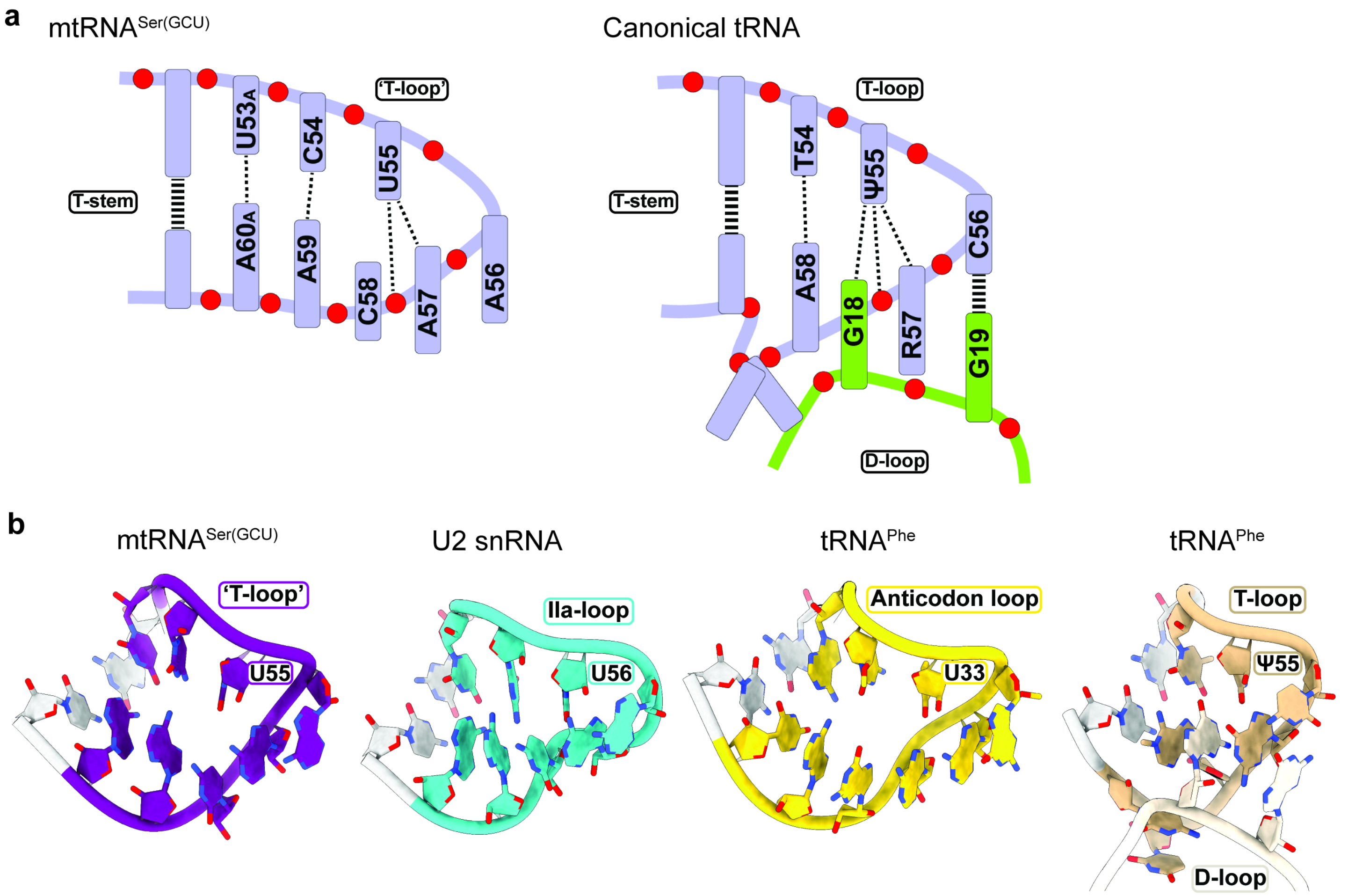

Supplementary Figure 8. Elbow structure comparison between human mtRNA$^{Ser(GCU)}$ and the canonical *S. cerevisiae* cytoplasmic tRNA^{Phe}. Related to Fig. 2. **a**. Schematic presentation of the T-loop/elbow region in mtRNA$^{Ser(GCU)}$ (left) and *S. cerevisiae* tRNA^{Phe} (right). **b**. Comparison of the 'T-loop' in human mtRNA$^{Ser(GCU)}$ with U-turns in the U2 snRNA (PDB 1U2A)[4], the anticodon loop, and the canonical T-loop in *S. cerevisiae* tRNA^{Phe} (PDB 4TRA). In each case, a conserved uridine, forming a stabilizing hydrogen bond with the phosphate moiety in the +3 position, is indicated (U55 in mtRNA$^{Ser(GCU)}$; modified to ψ in the T-loop of *S. cerevisiae* tRNA^{Phe}). In each structure, grey nucleotides correspond to the last (fifth) base-pair in the T-stem of canonical tRNAs on which the alignment is based. The wybutosine modification at position 37 in the anticodon loop of yeast tRNA^{Phe} was removed for clarity.

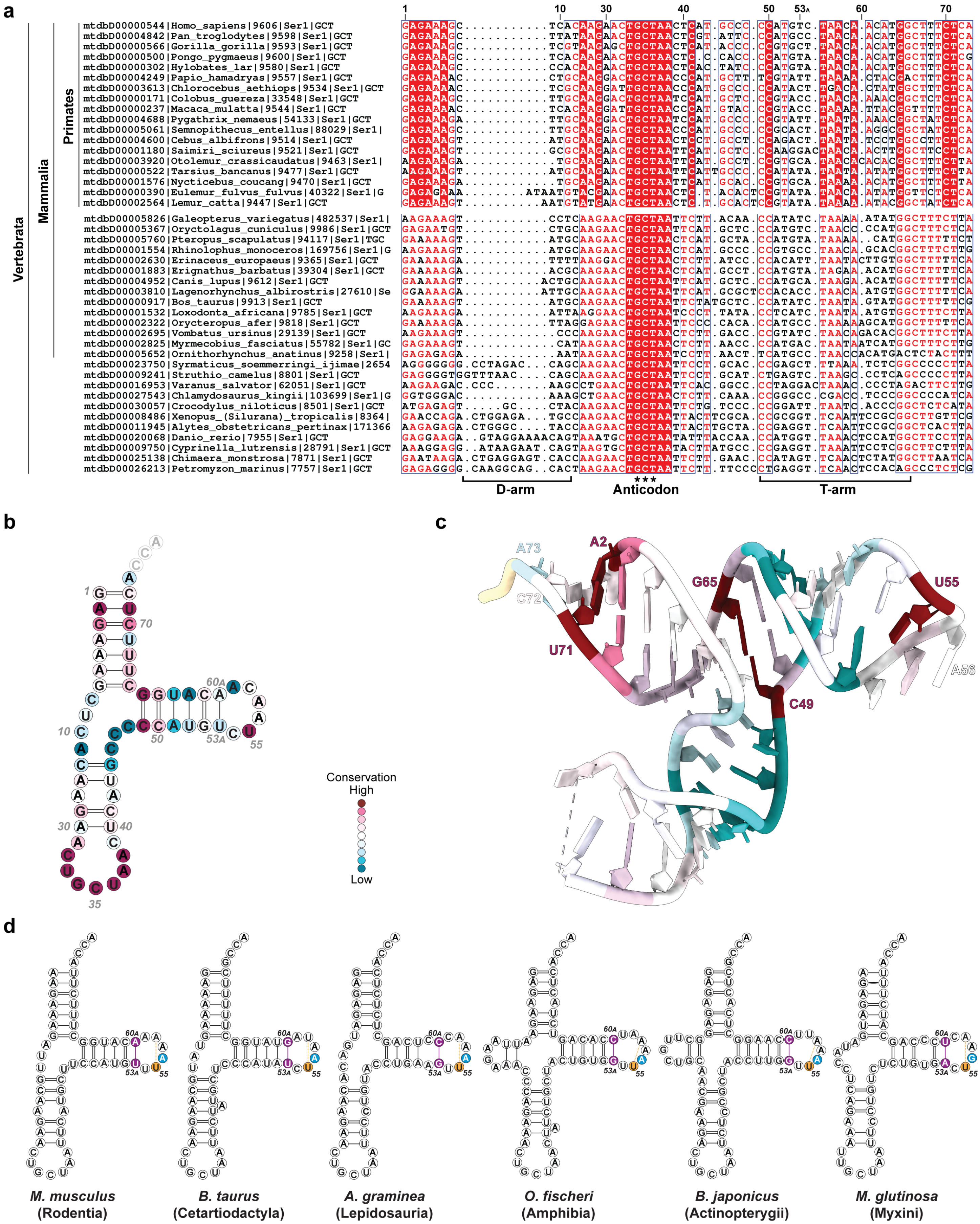
a
Vertebrata
Mammalia
Primates
mtdbD00000544|Homo_sapiens|9606|Ser1|GCT
mtdbD00004842|Pan_troglodytes|9598|Ser1|GCT
mtdbD00000566|Gorilla_gorilla|9593|Ser1|GCT
mtdbD00000500|Pongo_pygmaeus|9600|Ser1|GCT
mtdbD00000302|Hylobates_lar|9580|Ser1|GCT
mtdbD00004249|Papio_hamadryas|9557|Ser1|GCT
mtdbD00003613|Chlorocebus_aethiops|9534|Ser1|GCT
mtdbD00000171|Colobus_guereza|33548|Ser1|GCT
mtdbD00000237|Macaca_mulatta|9544|Ser1|GCT
mtdbD00004688|Pygathrix_nemaeus|54133|Ser1|GCT
mtdbD00005061|Semnopithecus_entellus|88029|Ser1|
mtdbD00004600|Cebus_albifrons|9514|Ser1|GCT
mtdbD00001180|Saimiri_sciureus|9521|Ser1|GCT
mtdbD00003920|Otolemur_crassicaudatus|9463|Ser1|
mtdbD00000522|Tarsius_bancanus|9477|Ser1|GCT
mtdbD00001576|Nycticebus_coucang|9470|Ser1|GCT
mtdbD00000390|Eulemur_fulvus_fulvus|40322|Ser1|G
mtdbD00002564|Lemur_catta|9447|Ser1|GCT
mtdbD00005826|Galeopterus_variegatus|482537|Ser1|
mtdbD00005367|Oryctolagus_cuniculus|9986|Ser1|GCT
mtdbD00005760|Pteropus_scapulatus|94117|Ser1|TGC
mtdbD00001554|Rhinolophus_monoceros|169756|Ser1|G
mtdbD00002630|Erinaceus_europaeus|9365|Ser1|GCT
mtdbD00001883|Erignathus_barbatus|39304|Ser1|GCT
mtdbD00004952|Canis_lupus|9612|Ser1|GCT
mtdbD00003810|Lagenorhynchus_albirostris|27610|Se
mtdbD00000917|Bos_taurus|9913|Ser1|GCT
mtdbD00001532|Loxodonta_africana|9785|Ser1|GCT
mtdbD00002322|Orycteropus_afer|9818|Ser1|GCT
mtdbD00002695|Vombatus_ursinus|29139|Ser1|GCT
mtdbD00002825|Myrmecobius_fasciatus|55782|Ser1|GC
mtdbD00005652|Ornithorhynchus_anatinus|9258|Ser1|
mtdbD00023750|Syrmaticus_soemmerringi_ijimae|2654
mtdbD00009241|Struthio_camelus|8801|Ser1|GCT
mtdbD00016953|Varanus_salvator|62051|Ser1|GCT
mtdbD00027543|Chlamydosaurus_kingii|103699|Ser1|G
mtdbD00030057|Crocodylus_niloticus|8501|Ser1|GCT
mtdbD00008486|Xenopus_(Silurana)_tropicalis|8364|
mtdbD00011945|Alytes_obstetricans_pertinax|171366
mtdbD00020068|Danio_rerio|7955|Ser1|GCT
mtdbD00009750|Cyprinella_lutrensis|28791|Ser1|GCT
mtdbD00025138|Chimaera_monstrosa|7871|Ser1|GCT
mtdbD00026213|Petromyzon_marinus|7757|Ser1|GCT
1 10 30 40 50 53A 60 70
D-arm
Anticodon
T-arm
b
Conservation
High
Low
c
A73
A2
G65
U55
C72
U71
A56
C49
d
M. musculus
(Rodentia)
B. taurus
(Cetartiodactyla)
A. graminea
(Lepidosauria)
O. fischeri
(Amphibia)
B. japonicus
(Actinopterygii)
M. glutinosa
(Myxini)

Supplementary Figure 9. Sequence and structure conservation of mtRNA$^{Ser(GCU)}$. Related to Figures 2-4. **a.** Multiple sequence alignment of vertebrate mtRNA$^{Ser(GCU)}$. Generated with Clustal omega (https://www.ebi.ac.uk/Tools/msa/clustalo/). **b**. Conservation of nucleotide positions among vertebrate mtRNA$^{Ser(GCU)}$ plotted onto the secondary structure of human mtRNA$^{Ser(GCU)}$. Conservation scores were calculated from the alignment of 950 vertebrate mtRNA$^{Ser(GCU)}$ sequences using the ConSurf server (https://consurf.tau.ac.il/). The non-coded 3'-CCA end was excluded. **c**. Conservation of nucleotide positions from **b** plotted onto the tertiary structure of human mtRNA$^{Ser(GCU)}$. The color code is the same as in **b**. **d**. Secondary structures of vertebrate mtRNA$^{Ser(GCU)}$ variants showing the conservation of the unusual T-arm structural features found in human mtRNA$^{Ser(GCU)}$. The additional T-stem pair (53_A:60_A) is highlighted in purple, U55 is highlighted in orange, and the R56, which replaces the highly conserved canonical C56 at the tip of the U-turn, is highlighted in blue.

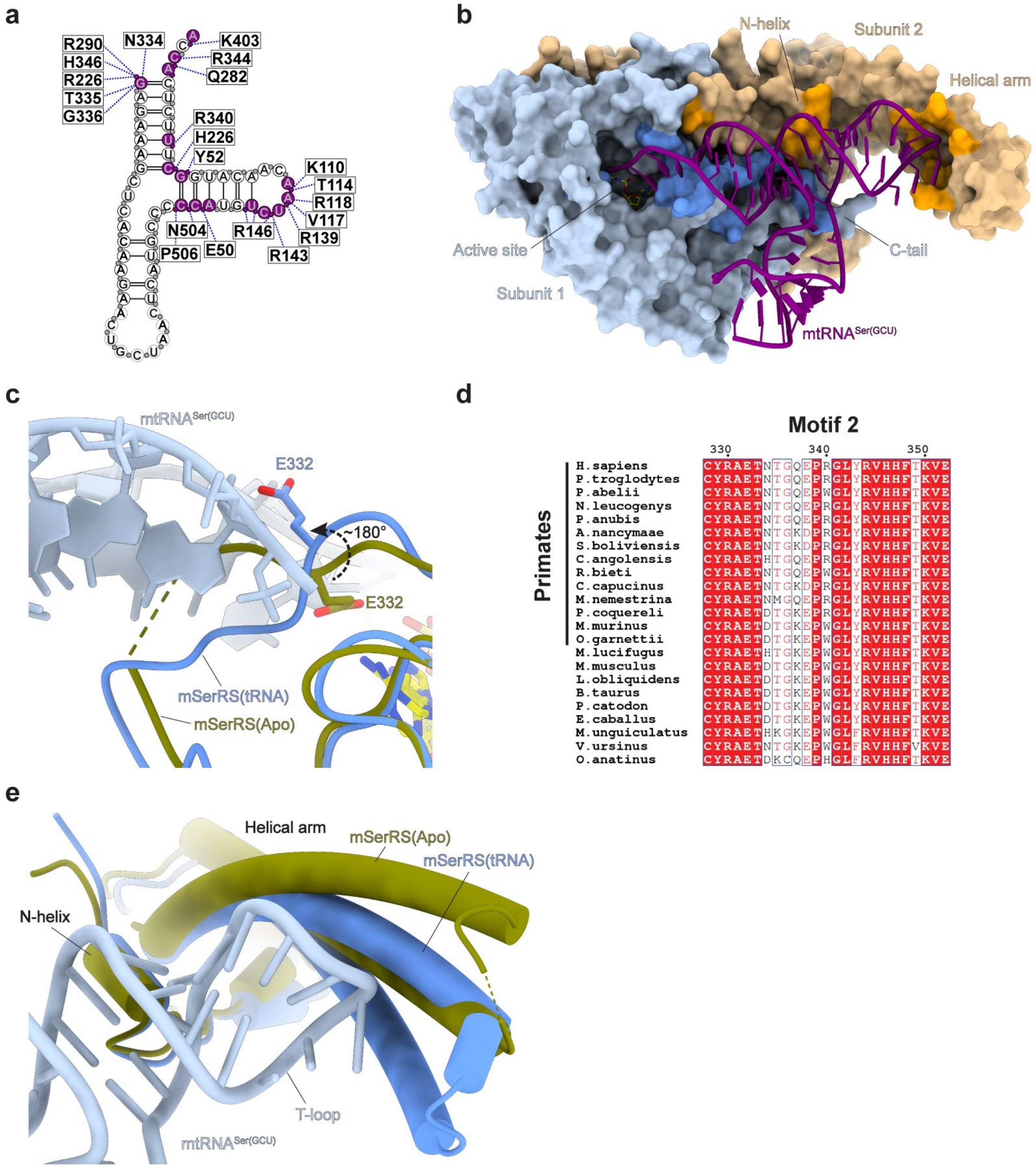

Supplementary Figure 10. Interfaces and conformational rearrangements in the mSerRS-mtRNA$^{Ser(GCU)}$ complex. Related to Figures 3 and 4. **a.** Summary of interactions between mtRNA$^{Ser(GCU)}$ and mSerRS. Interactions are formed either with the base (large circles) or the sugar-phosphate backbone (small connecting circles) of the tRNA. **b**. mtRNA$^{Ser(GCU)}$ binding interface on mSerRS. Interfaces on the mSerRS dimer surface are highlighted in blue and orange for subunits 1 and 2, respectively. **c**. Detail of conformational rearrangements in the motif 2 loop upon tRNA binding, including a ~180° switch of E332. Apo mSerRS is shown in green, tRNA-bound mSerRS and tRNA are shown in blue and light blue, respectively. **d.** Multiple sequence alignment of motif 2 in mammalian mSerRSs. Generated with Clustal omega (https://www.ebi.ac.uk/Tools/msa/clustalo/). **e**. Conformational rearrangements in the mSerRS helical arm upon tRNA binding. Coloring is the same as in **c**.

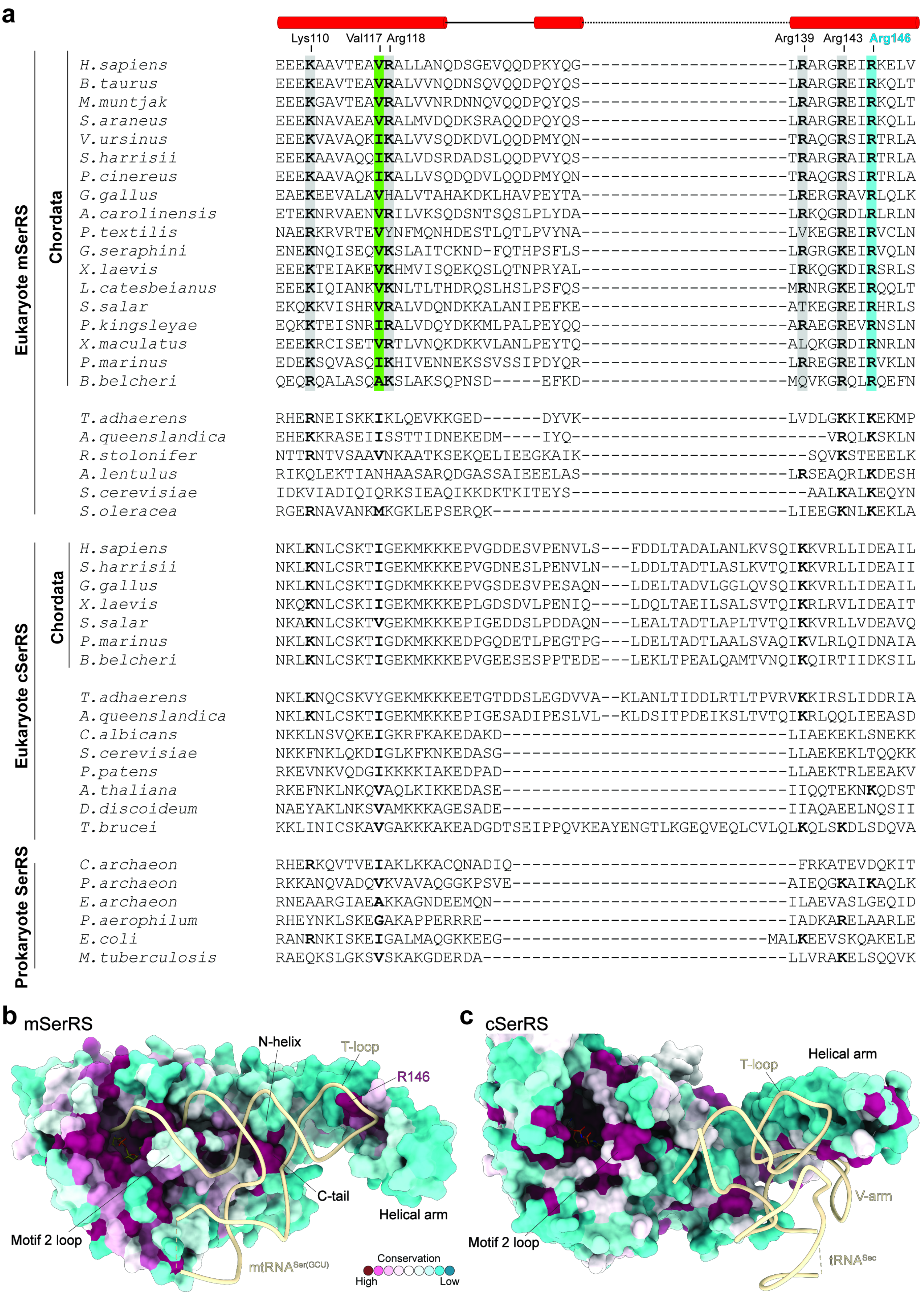

a
Lys110
Val117
Arg118
Arg139
Arg143
Arg146
Eukaryote mSerRS
Chordata
H.sapiens EEEKAAVTEAVRALLANQDSGEVQQDPKYQG---------------------LRARGREIRKELV
B.taurus EEEKEAVTEAVRALVVNQDNSQVQQDPQYQS---------------------LRARGREIRKQLT
M.muntjak EEEKGAVTEAVRALVVNRDNNQVQQDPQYQS---------------------LRARGREIRKQLT
S.araneus EEEKNAVAEAVRALMVDQDKSRAQQDPQYQS---------------------LRARGREIRKQLL
V.ursinus EEEKVAVAQKIKALVVSQDKDVLQQDPMYQN---------------------TRAQGRSIRTRLA
S.harrisii EEEKAAVAQQIKALVDSRDADSLQQDPVYQS---------------------TRARGRAIRTRLA
P.cinereus EEEKAAVAQKIKALLVSQDQDVLQQDPMYQN---------------------TRAQGRSIRTRLA
G.gallus EAEKEEVALAVHALVTAHAKDKLHAVPEYTA---------------------LRERGRAVRLQLK
A.carolinensis ETEKNRVAENVRILVKSQDSNTSQSLPLYDA---------------------LRKQGRDLRLRLN
P.textilis NAERKRVRTEVYNFMQNHDESTLQTLPVYNA---------------------LVKEGREIRVCLN
G.seraphini ENEKNQISEQVKSLAITCKND-FQTHPSFLS---------------------LRGRGKEIRVQLN
X.laevis EEEKTEIAKEVKHMVISQEKQSLQTNPRYAL---------------------IRKQGKDIRSRLS
L.catesbeianus EEEKIQIANKVKNLTLTHDRQSLHSLPSFQS---------------------MRNRGKEIRQQLT
S.salar EKQKKVISHRVRALVDQNDKKALANIPEFKE---------------------ATKEGREIRHRLS
P.kingsleyae EQKKTEISNRIRALVDQYDKKMLPALPEYQQ---------------------ARAEGREVRNSLN
X.maculatus EEEKRCISETVRTLVNQKDKKVLANLPEYTQ---------------------ALQKGRDIRNRLN
P.marinus EDEKSQVASQIKHIVENNEKSSVSSIPDYQR---------------------LRREGREIRVKLN
B.belcheri QEQRQALASQAKSLAKSQPNSD-----EFKD---------------------MQVKGRQLRQEFN
T.adhaerens RHERNEISKKIKLQEVKKGED------DYVK---------------------LVDLGKKIKEKMP
A.queenslandica EHEKKRASEIISSTTIDNEKEDM----IYQ--------------------------VRQLKSKLN
R.stolonifer NTTRNTVSAAVNKAATKSEKQELIEEGKAIK-----------------------SQVKSTEEELK
A.lentulus RIKQLEKTIANHAASARQDGASSAIEEELAS---------------------LRSEAQRLKDESH
S.cerevisiae IDKVIADIQIQRKSIEAQIKKDKTKITEYS------------------------AALKALKEQYN
S.oleracea RGERNAVANKMKGKLEPSERQK------------------------------LIEEGKNLKEKLA
Eukaryote cSerRS
Chordata
H.sapiens NKLKNLCSKTIGEKMKKKEPVGDDESVPENVLS---FDDLTADALANLKVSQIKKVRLLIDEAIL
S.harrisii NKLKNLCSRTIGEKMKKKEPVGDNESLPENVLN---LDDLTADTLASLKVTQIKKVRLLIDEAII
G.gallus NKLKNLCSKTIGDKMKKKEPVGSDESVPESAQN---LDELTADVLGGLQVSQIKKVRLLIDEAIL
X.laevis NKQKNLCSKIIGEKMKKKEPLGDSDVLPENIQ----LDQLTAEILSALSVTQIKRLRVLIDEAIT
S.salar NKAKNLCSKTVGEKMKKKEPIGEDDSLPDDAQN---LEALTADTLAPLTVTQIKKVRLLVDEAVQ
P.marinus NKLKNLCSKTIGDKMKKKEDPGQDETLPEGTPG---LDELTADTLAALSVAQIKVLRLQIDNAIA
B.belcheri NRLKNLCSKTIGEKMKKKEPVGEESESPPTEDE---LEKLTPEALQAMTVNQIKQIRTIIDKSIL
T.adhaerens NKLKNQCSKVYGEKMKKKEETGTDDSLEGDVVA--KLANLTIDDLRTLTPVRVKKIRSLIDDRIA
A.queenslandica NKLKNLCSKTIGEKMKKKEPIGESADIPESLVL--KLDSITPDEIKSLTVTQIKRLQQLIEEASD
C.albicans NKKLNSVQKEIGKRFKAKEDAKD-----------------------------LIAEKEKLSNEKK
S.cerevisiae NKKFNKLQKDIGLKFKNKEDASG-----------------------------LLAEKEKLTQQKK
P.patens RKEVNKVQDGIKKKKIAKEDPAD-----------------------------LLAEKTRLEEAKV
A.thaliana RKEFNKLNKQVAQLKIKKEDASE-----------------------------IIQQTEKNKQDST
D.discoideum NAEYAKLNKSVAMKKKAGESADE-----------------------------IIAQAEELNQSII
T.brucei KKLINICSKAVGAKKKAKEADGDTSEIPPQVKEAYENGTLKGEQVEQLCVLQLKQLSKDLSDQVA
Prokaryote SerRS
C.archaeon RHERKQVTVEIAKLKKACQNADIQ---------------------------FRKATEVDQKIT
P.archaeon RKKANQVADQVKVAVAQGGKPSVE----------------------------AIEQGKAIKAQLK
E.archaeon RNEAARGIAEAKKAGNDEEMQN------------------------------ILAEVASLGEQID
P.aerophilum RHEYNKLSKEGAKAPPERRRE-------------------------------IADKARELAARLE
E.coli RANRNKISKEIGALMAQGKKEEG---------------------------MALKEEVSKQAKELE
M.tuberculosis RAEQKSLGKSVSKAKGDERDA-------------------------------LLVRAKELSQQVK
b mSerRS
N-helix
T-loop
R146
C-tail
Helical arm
Motif 2 loop
mtRNASer(GCU)
Conservation
High
Low
c cSerRS
T-loop
Helical arm
V-arm
Motif 2 loop
tRNASec

Supplementary Figure 11. Conservation of the mtRNA$^{Ser(GCU)}$ binding interface in mSerRS. Related to Fig. 4. **a.** Sequence alignment of the helical arm from prokaryotic, eukaryote cytoplasmic (c), and mitochondrial (m) SerRSs. Residues forming the helical arm interface are indicated. The catalytically important Arg146 is highlighted in cyan. **b.** Sequence conservation in vertebrate mitochondrial SerRSs plotted onto the mSerRS-mtRNA$^{Ser(GCU)}$ complex structure. Conservation scores were calculated from the alignment of 213 vertebrate mSerRS sequences using the ConSurf server (https://consurf.tau.ac.il/). The tRNA is shown in cartoon presentation in wheat. **c.** Sequence conservation in eukaryote cytoplasmic SerRSs plotted onto the human cytoplasmic SerRS-tRNA^{Sec} complex structure (PDB 4RQF)[5]. Conservation scores were calculated from the alignment of 11 eukaryote cytoplasmic SerRS sequences using the ConSurf server (https://consurf.tau.ac.il/). The tRNA is shown in cartoon presentation in wheat. Comparison with **b** shows the shift in highly conserved residues from the 'helical arm pit' which recognizes the long V-arm in canonical SerRSs to the helical arm binding pocket in mSerRSs, which recognizes the idiosyncratic 'T-loop' of mtRNA$^{Ser(GCU)}$.

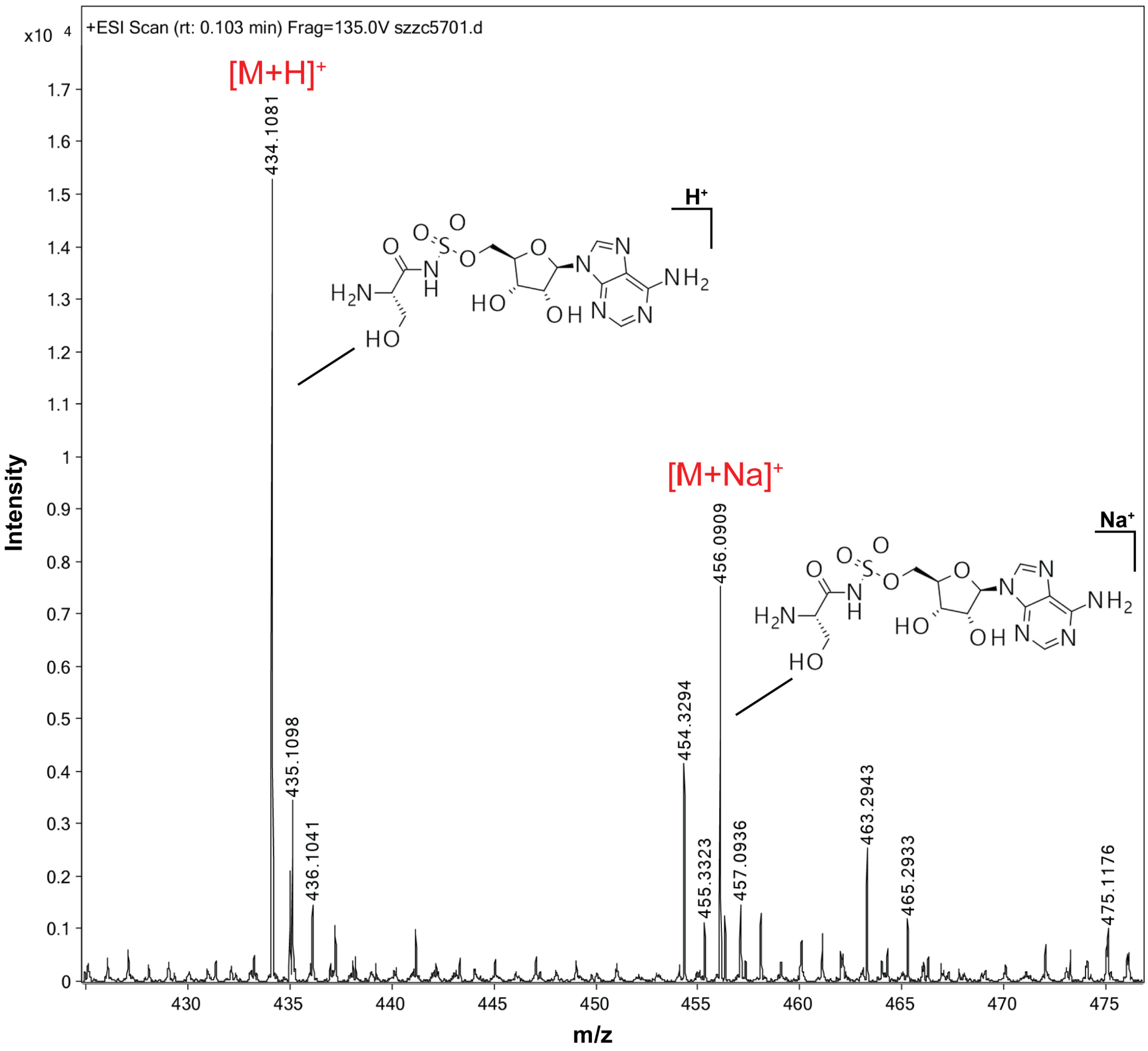

Supplementary Figure 12. ESI-TOF mass spectrum of 5'-O-[N-(L-seryl)sulfamoyl] adenosine (SerSA), purified as described in the Methods section. The peaks at m/z 434.1081 and m/z 456.0909 correspond to the protonated $[SerSA+H]^+$ ion and $[SerSA+Na]^+$ ion, respectively. The molecular mass of SerSA ($C_{13}H_{19}N_7O_8S$) is 433.1016 Da.

Supplementary Table 1.
X-ray crystallographic data collection and refinement statistics (related to Fig. 1).

**Data Collection**	**mSerRS-SerSA**
Radiation source	SSRL BL 12-2
Wavelength (Å)	0.9795
Space group	*R*3
Protein mol/AU	2
Cell dimensions	a=b=150.59 Å
	c=142.47 Å
	α=β=90°
	γ=120°
Resolution (Å)	37.65–2.95 (3.13–2.95)
Total reflections	120232 (20117)
Unique reflections	46505 (7988)
Completeness (%)	91.4 (97.4)
Redundancy	2.8 (2.5)
R_{meas} (%)	6.7 (88.6)
I/σ(I)	10.9 (1.3)
$CC_{1/2}$	99.8 (75.3)
Wilson B-factor (Å^2)	98.3
**Refinement**	
Refinement package	Phenix-1.20.1
Search model (PDB ID)	1WLE
Resolution (Å)	37.65–2.95
$Reflections_{Work}$	23445
$Reflections_{Test}$	1172
R_{work} (%)	24.04
R_{free} (%)	26.69
No. of atoms	
Protein	7324
Ligand	58
Water	0
Ions	0
Average overall B-factor (Å^2)	123.66
Average B-factors protein/ligands (Å^2)	123.84/100.12
Rmsd bond lengths (Å)	0.013
Rmsd bond angles (°)	1.765
Ramachandran statistics (MolProbity)	
favored (%)	99.34
outliers (%)	0.0
Rotamer outliers (%)	0.39
Clash score	11.17
MolProbity score	1.56
PDB deposition ID	7TZB

Values in parentheses refer to the highest resolution shell. The structure was determined from one crystal.

Supplementary Table 2.
Cryo-EM data collection, refinement, and validation statistics (related to Fig. 1).

	**mSerRS-mtRNA$^{Ser(GCU)-TL}$**	**mSerRS-mtRNA$^{Ser(GCU)}$**
PDB ID<br>EMDB ID	7U2B<br>26311	7U2A<br>26310
**Data collection and processing**		
Microscope	Talos Arctica	Talos Arctica
Voltage (keV)	200	200
Nominal magnification	X36,000	X36,000
Exposure navigation	Image shift	Image shift
Electron exposure (e^- Å^{-2})	66	66
Exposure rate (e^- pixel^{-1} s^{-1})	5.5	5.5
Detector	K2 Summit	K2 Summit
Defocus range (µm)	-0.8 to -1.2	-0.8 to -1.2
Pixel size (Å)	1.15	1.15
Micrographs	1330	2498
Automation software	Leginon	Leginon
Total extracted particles (no.)	1,401,961	1,049,173
Refined particles (no.)	1,401,961	1,049,173
**Reconstruction**		
Final particles (no.)	118,269	152,066
Symmetry imposed	C1	C1
Map resolution (Å)<br>FSC 0.5 (unmasked/masked)<br>FSC 0.143 (unmasked/masked)	4.1<br>4.9/4.1<br>4.3/4.1	4.1<br>4.7/4.1<br>4.3/4.1
Map resolution range (Å)	3.8-6.2	3.8-6.2
Applied B-factor (Å^2)	-150	-150
3D FSC Sphericity (%)	0.882	0.843
**Refinement**		
Refinement package	Phenix-1.20.1	Phenix-1.20.1
Initial model used (PDB code)	mSerRS (7TZB)	mSerRS-mtRNA$^{Ser(GCU)-TL}$
Model composition<br>Protein residues<br>Nucleotide residues	 780<br>53	 761<br>38
Map Correlation Coefficient	0.67	0.63
Average *B* factors (Å^2)<br>Protein<br>tRNA	 130<br>47	 126<br>48
R.m.s. deviations<br>Bond lengths (Å)<br>Bond angles (°)	 0.006<br>0.842	 0.007<br>0.933
Ramachandran plot<br>Favored (%)<br>Allowed (%)<br>Disallowed (%)	 99.87<br>0.13<br>0.0	 99.47<br>0.53<br>0.0
Poor rotamers (%)	0.15	0.31
MolProbity score	1.39	1.56
Clashscore (all atoms)	7.07	10.95
C-beta deviations	0.0	0.0
CaBLAM outliers (%)	0.79	0.81
EMRinger score	0.51	0.62

Supplementary Table 3.

Kinetics of aminoacylation of mtRNA$^{Ser(GCU)}$ variants by mSerRS (related to Fig. 5).

tRNA variants	k_{obs} (10^{-3} s^{-1})	k_{obs} *relative*
WT ('GC')	83.2 ± 0.8	1.00
A73G	92.8 ± 5.0	1.12
A42U	89.7 ± 0.5	1.08
2xGC	88.4 ± 3.4	1.06
U55C	22.6 ± 2.8	0.27
A56G	111.6 ± 6.5	1.34
A56C	38.1 ± 1.3	0.46
A57G	82.8 ± 10.8	1.00
A57C	21.3 ± 2.6	0.26
T-loop	7.5 ± 0.5	0.09
ΔU52:A62	6.8 ± 1.7	0.08

Means and standard errors were calculated from three independent experiments.

Supplementary Table 4.
Kinetics of aminoacylation of $mtRNA^{Ser(GCU)}$ by mSerRS mutants (related to Fig. 5).

mSerRS	k_{cat} (min^{-1})	K_m μM	k_{cat}/K_m (min^{-1} μM^{-1})	k_{cat}/K_m relative
WT	8.85 ± 0.21	1.97 ± 0.09	4.50	1.00
K110A	8.30 ± 0.06	1.46 ± 0.02	5.70	1.27
R118A	8.38 ± 0.43	2.43 ± 0.30	3.45	0.77
R139A	9.28 ± 0.40	3.41 ± 0.21	2.72	0.60
R143A	8.12 ± 0.13	3.47 ± 0.02	2.34	0.52
3xRA	6.86 ± 0.56	9.81 ± 0.95	0.70	0.16
R146A	7.40 ± 0.84	11.98 ± 2.02	0.62	0.14

Means and standard errors were calculated from three independent experiments.

Supplementary Table 5.

Primers used in this study.

Human SARS2 PCR primer 5’<br>GGGGCCCCTGGGATCCACTACAGAGAAACGAAACCGGAACC
Human SARS2 PCR primer 3’<br>GATGCGGCCGCTCGAGTTAGCTTACAGCAGGCTGGCCAG
Human mtRNA$^{Ser(GCU)}$ PCR primer 5‘<br>CTGCTAACTCATGCCCCCATGTCTAACAACATGGCTTTCTCACCATAGAGGATCC
Human mtRNA$^{Ser(GCU)}$ PCR primer 3‘<br>GGCATGAGTTAGCAGTTCTTGTGAGCTTTCTCTATAGTGAGTCGTATTAATTAC
Human mtRNA$^{Ser(GCU)-TL}$ PCR primer 5‘<br>ACAAGCGAAACCATGCCCCCATGTCTAACAACATGGCTTTC
Human mtRNA$^{Ser(GCU)-TL}$ PCR primer 3‘<br>GCATGGTTTCGCTTGTGAGCTTTCTCTATAGTGAGTCGTATTAATTAC
T7-promoter template PCR primer 5‘<br>GGATCCTAATACGACTCACTATAG
Human mtRNA$^{Ser(GCU)}$ template PCR primer 3‘<br>TGGTGAGAAAGCCATGTTGTTAGACATG
Human mtRNA$^{Ser(GCU)}$ (‘GC’) template PCR primer 3‘<br>TGGTGGGAAAGCCATGTTGTTAGAC

Supplementary References

1 Sprinzl, M., Horn, C., Brown, M., Ioudovitch, A. & Steinberg, S. Compilation of tRNA sequences and sequences of tRNA genes. *Nucleic Acids Res* **26**, 148-153, doi:10.1093/nar/26.1.148 (1998).

2 Westhof, E., Dumas, P. & Moras, D. Restrained refinement of two crystalline forms of yeast aspartic acid and phenylalanine transfer RNA crystals. *Acta Crystallogr A* **44 (Pt 2)**, 112-123 (1988).

3 Chimnaronk, S., Gravers Jeppesen, M., Suzuki, T., Nyborg, J. & Watanabe, K. Dual-mode recognition of noncanonical tRNAs(Ser) by seryl-tRNA synthetase in mammalian mitochondria. *EMBO J* **24**, 3369-3379, doi:10.1038/sj.emboj.7600811 (2005).

4 Stallings, S. C. & Moore, P. B. The structure of an essential splicing element: stem loop IIa from yeast U2 snRNA. *Structure* **5**, 1173-1185, doi:10.1016/s0969-2126(97)00268-2 (1997).

5 Wang, C. *et al.* SerRS-tRNASec complex structures reveal mechanism of the first step in selenocysteine biosynthesis. *Nucleic Acids Res* **43**, 10534-10545, doi:10.1093/nar/gkv996 (2015).
