# Supplementary material for: Structural basis for shape-selective recognition and aminoacylation of a D-armless human mitochondrial tRNA
Source: Nat Commun. 2022 Aug 30;13:5100. doi: 10.1038/s41467-022-32544-1 (PMC9427863; doi:10.1038/s41467-022-32544-1)
Supplement: Supplementary file 3 — Reporting Summary [file 41467_2022_32544_MOESM3_ESM.pdf]

Corresponding author(s): Bernhard Kuhle

Last updated by author(s): Aug 1, 2022

Reporting Summary

Nature Portfolio wishes to improve the reproducibility of the work that we publish. This form provides structure for consistency and transparency in reporting. For further information on Nature Portfolio policies, see our Editorial Policies and the Editorial Policy Checklist.

Statistics

For all statistical analyses, confirm that the following items are present in the figure legend, table legend, main text, or Methods section.

n/a	Confirmed	
☐	☒	The exact sample size (*n*) for each experimental group/condition, given as a discrete number and unit of measurement
☒	☐	A statement on whether measurements were taken from distinct samples or whether the same sample was measured repeatedly
☒	☐	The statistical test(s) used AND whether they are one- or two-sided<br>*Only common tests should be described solely by name; describe more complex techniques in the Methods section.*
☒	☐	A description of all covariates tested
☒	☐	A description of any assumptions or corrections, such as tests of normality and adjustment for multiple comparisons
☐	☒	A full description of the statistical parameters including central tendency (e.g. means) or other basic estimates (e.g. regression coefficient) AND variation (e.g. standard deviation) or associated estimates of uncertainty (e.g. confidence intervals)
☒	☐	For null hypothesis testing, the test statistic (e.g. *F*, *t*, *r*) with confidence intervals, effect sizes, degrees of freedom and *P* value noted<br>*Give P values as exact values whenever suitable.*
☒	☐	For Bayesian analysis, information on the choice of priors and Markov chain Monte Carlo settings
☒	☐	For hierarchical and complex designs, identification of the appropriate level for tests and full reporting of outcomes
☒	☐	Estimates of effect sizes (e.g. Cohen's *d*, Pearson's *r*), indicating how they were calculated

Our web collection on statistics for biologists contains articles on many of the points above.

Software and code

Policy information about availability of computer code

Data collection	Cryo-EM data collection was automated using the Leginon data collection software (Suloway et al. J Struct Biol, 2005).
Data analysis	Kinetic data were analyzed using GraphPad Prism 8 (GraphPad Software, Inc.)<br>Mass Photometry data were analyzed using DiscoverMP software (Refeyn Ltd, Oxford, UK).<br>Sequence alignments were done using the Molecular Evolutionary Genetics Analysis (MEGA 7.0) software (Kumar et al. Mol Biol Evol, 2016).<br>X-ray diffraction images were processed using the XDS package (Kabsch, Acta Crystallogr D Biol Crystallogr., 2010).<br>Cryo-EM data were analyzed using Warp (Tegunov & Cramer, Nat Methods, 2019), CryoSPARC (Punjani et al., Nat. Methods, 2017), MotionCor2 (Zheng et al., Nat. Methods, 2017), RELION 3.1 (Zivanov et al., Elife, 2018), Gctf (Zhang, J. Struct. Biol., 2016), and the the 3D FSC server (Tan et al., Nat Methods, 2017).<br>Molecular replacement (X-ray crystallography), model building, and refinement were performed using Coot and Phenix-1.10.1 (Adams et al., Acta Crystallogr D Biol Crystallogr., 2010; Emsley et al., Acta Crystallogr D Biol Crystallogr., 2010), and the Rosetta Online Server (https://rosie.graylab.jhu.edu/).<br>Structures were visualized and analyzed in ChimeraX (Goddard et al., Protein Sci., 2018).

For manuscripts utilizing custom algorithms or software that are central to the research but not yet described in published literature, software must be made available to editors and reviewers. We strongly encourage code deposition in a community repository (e.g. GitHub). See the Nature Portfolio guidelines for submitting code & software for further information.

Data

Policy information about availability of data

All manuscripts must include a data availability statement. This statement should provide the following information, where applicable:

- Accession codes, unique identifiers, or web links for publicly available datasets
- A description of any restrictions on data availability
- For clinical datasets or third party data, please ensure that the statement adheres to our policy

All data generated or analyzed during this study are included in this published article (and its supplementary information files). Cryo-EM maps of mSerRS-mtRNASer (GCU) and mSerRS-mtRNASer(GCU)-TL have been deposited in the Electron Microscopy Data Bank (EMDB) under the accession codes EMD-26310 and EMD-26311, respectively. Atomic coordinates of the models have been deposited in the Protein Data Bank (PDB) under accession codes 7U2A, 7U2B, and 7TZB for the crystal structure of mSerRS bound to SerSA. The atomic coordinates used for molecular replacement or structural comparison were downloaded from the Protein Data Bank: 1SER, 1U2A, 1WLE, 3L0U, 4RDX, 4RQF, 4TRA, and 6UGG. tRNA gene sequences were retrieved from tRNAdb/mitotRNAdb (http://trna.bioinf.uni-leipzig.de/) and the genomic tRNA database (GtRNAdb; http://gtrnadb.ucsc.edu/). The data underlying Fig. 5a and 5c have been deposited as a Source Data file with this paper.

Field-specific reporting

Please select the one below that is the best fit for your research. If you are not sure, read the appropriate sections before making your selection.

☒ Life sciences ☐ Behavioural & social sciences ☐ Ecological, evolutionary & environmental sciences

For a reference copy of the document with all sections, see nature.com/documents/nr-reporting-summary-flat.pdf

Life sciences study design

All studies must disclose on these points even when the disclosure is negative.

Sample size	Sample sizes were not predetermined using statistical methods. For cryo-EM structure determination, the total number of movies for the tRNASer(GCU)-TL and tRNASer(GCU) datasets were 2498 and 1330, respectively. The resulting particle stacks contained 3.5M particles for the tRNASer(GCU) dataset and 6.9M particles for the tRNASer(GCU)-TL dataset. The adequacy of the sample size was judged by the resulting cryo-EM maps, which were of sufficiently high quality for structural fitting and atomic model building. The in vitro kinetic experiments were repeated three times, which is comparable to other published studies and allowed statistical analysis.
Data exclusions	No data were excluded from our analyses.
Replication	In vitro experiments were repeated three times and all replication attempts were successful.
Randomization	This study did not include treatment groups.
Blinding	Due to the nature of the project (understand molecular function of human mitochondrial aaRS/tRNA systems), investigators were not blinded and were aware of the sequence characteristics of all proteins/tRNAs used in the reported experiments.

Reporting for specific materials, systems and methods

We require information from authors about some types of materials, experimental systems and methods used in many studies. Here, indicate whether each material, system or method listed is relevant to your study. If you are not sure if a list item applies to your research, read the appropriate section before selecting a response.

Materials & experimental systems

n/a	Involved in the study
☒	☐ Antibodies
☒	☐ Eukaryotic cell lines
☒	☐ Palaeontology and archaeology
☒	☐ Animals and other organisms
☒	☐ Human research participants
☒	☐ Clinical data
☒	☐ Dual use research of concern

Methods

n/a	Involved in the study
☒	☐ ChIP-seq
☒	☐ Flow cytometry
☒	☐ MRI-based neuroimaging
